# Investigation of two suspected diarrhoeal-illness outbreaks in Northern Cape and KwaZulu-Natal provinces, South Africa, April–July 2013: The role of rotavirus

**DOI:** 10.4102/sajid.v35i1.159

**Published:** 2020-07-22

**Authors:** Andronica M. Shonhiwa, Genevie Ntshoe, Noreen Crisp, Ayo J. Olowolagba, Vusi Mbuthu, Maureen B. Taylor, Juno Thomas, Nicole A. Page

**Affiliations:** 1 Division of Public Health Surveillance and Response, National Institute for Communicable Diseases, National Health Laboratory Service, Sandringham, Johannesburg, South Africa; 2 School of Health Systems and Public Health, Faculty of Health Science, University of Pretoria, Pretoria, South Africa; 3 Communicable Disease Control, Department of Health, Kimberley, South Africa; 4 Communicable Disease Control, eThekwini Metropolitan Municipality Department of Health, Durban, South Africa; 5 Department of Medical Virology, Faculty of Health Sciences, University of Pretoria, Pretoria, South Africa; 6 National Health Laboratory Service, Tshwane Academic Division, Pretoria, South Africa; 7 Centre for Enteric Diseases, National Institute for Communicable Diseases, National Health Laboratory Service, Sandringham, Johannesburg, South Africa

**Keywords:** diarrhoeal illness, outbreak, rotavirus, rotavirus vaccine, South Africa

## Abstract

**Background:**

Suspected diarrhoeal-illness outbreaks affecting mostly children < 5 years were investigated between May and July 2013 in Northern Cape province (NCP) and KwaZulu-Natal (KZN) province. This study describes the epidemiological, environmental and clinical characteristics and diarrhoeal-illnesses causative agent(s).

**Methods:**

A descriptive cross-sectional study was conducted. Cases were patients presenting at healthcare facilities with diarrhoeal-illness between 09 April and 09 July 2013 in NCP and 01 May and 31 July 2013 in KZN. Laboratory investigations were performed on stools and water samples using microscopy, culture and sensitivity screening and molecular assays.

**Results:**

A total of 953 cases including six deaths (case fatality rate [CFR]: 0.6%) were recorded in the Northern Cape province outbreak. Children < 5 years accounted for 58% of cases. Enteric viruses were detected in 51% of stools, with rotavirus detected in 43%. The predominant rotavirus strains were G3P[8] (45%) and G9P[8] (42%). Other enteric viruses were detected, with rotavirus co-infections (63%). No enteric pathogens detected in water specimens. *KwaZulu-Natal outbreak:* A total of 1749 cases including 26 deaths (CFR: 1.5%) were recorded. Children < 5 years accounted for 95% of cases. Rotavirus was detected in 55% of stools; other enteric viruses were detected, mostly as rotavirus co-infections. The predominant rotavirus strains were G2P[4] (54%) and G9P[8] (38%).

**Conclusion:**

Although source(s) of the outbreaks were not identified, the diarrhoeal-illnesses were community-acquired. It is difficult to attribute the outbreaks to one causative agent(s) because of rotavirus co-infections with other enteric pathogens. While rotavirus was predominant, the outbreaks coincided with the annual rotavirus season.

## Introduction

Diarrhoeal-illness is among the leading causes of morbidity and mortality among young children < 5 years, the elderly and patients with underlying medical conditions worldwide.^1,2,3,4,5^ Globally, more than 1 billion episodes of diarrhoea because of infectious pathogens occur in children < 5 years causing more than half a million deaths annually, with more deaths occurring in low-income countries in sub-Saharan Africa, South America and South Asia.^5,6,7,8,9^ In South Africa, acute diarrhoeal-illnesses are ranked the third leading cause of childhood mortality in children < 5 years.^10^

A study conducted in low-income countries to estimate pathogen-specific burdens of diarrhoea in children aged 0–24 months reported the highest attributable fractions for norovirus genogroup II (GII), rotavirus, *Campylobacter* species (spp.), astrovirus, *Cryptosporidium* spp. and *Shigella* spp. in the first and second years of life.^8^ Enteric bacteria and parasites along with rotavirus were found to be common causes of diarrhoea in children < 5 years.^11^ The most common bacterial pathogens causing diarrhoea were diarrhoeagenic *Escherichia coli* (48%), *Shigella* spp. (8%), *Salmonella* spp. (4%) and *Campylobacter* spp. (2%). Rotavirus was found in 22% and enteric parasites (*Giardia intestinalis* and *Entamoeba histolytica*) in 16% of children with diarrhoea.^11^ Almost 43% of stool specimens from children with diarrhoea, screened using molecular techniques, identified at least one viral agent.^12,13,14^ An estimated 40% of acute diarrhoeal-illness cases in children < 5 years are because of rotavirus infections, with approximately 30% because of norovirus and adenovirus infections.^12^ An additional 20% of diarrhoeal cases can be attributed to bacterial infections, 5% to parasites and the remaining 5% to other causes.^15^

Rotavirus is the leading cause of severe acute diarrhoea and death in children < 5 years worldwide.^16,17^ Global estimated deaths because of rotavirus in children < 5 years have declined from 528 000 (range: 465 000–591 000) in 2000 to 215 000 (197 000–233 000) in 2013. Sub-Saharan Africa accounted for the highest number of deaths because of rotavirus, 250 000 (range: 217 000–282 000) in 2000 declining to 121 000 (111 000–131 000) in 2013.^17^ More than 90% of rotavirus associated deaths in children < 5 years occurred in Africa and Asia.^16,18,19^ In South Africa, gastroenteritis was the leading cause of death (18%) in children < 5 years in 2009,^20^ and rotavirus was detected in approximately 25% of cases hospitalised because of diarrhoea.^10,19,21^ The burden of diarrhoeal-illness studies found that the majority (90%) of children < 24 months admitted or visiting an outpatient department for diarrhoea treatment were infected with rotavirus.^22,23^

Rotavirus vaccines have been introduced into the immunisation programmes of many countries and are expected to have an impact on rotavirus diarrhoea in children.^24^ In 2013, the World Health Organization (WHO) global vaccine coverage data showed that approximately 52 countries introduced the rotavirus vaccine.^25^ By the end of 2015, a further 32 countries had introduced the vaccine and the vaccine coverage was estimated to be 23% globally.^26^ South Africa introduced a monovalent (G1P[8]) rotavirus vaccine (Rotarix^®^, GlaxoSmithKline), with a 61% vaccine effectiveness, into the national expanded programme on immunisation (EPI) in August 2009.^10,19^ Rotarix^®^ vaccine studies conducted in South Africa reported 77% protective efficacy against severe diarrhoea in children < 5 years.^27,28,29,30,31^ Rotarix^®^ was well tolerated and immunogenic in human immunodeficiency virus (HIV)-infected infants.^31^ The vaccine is given in a two-dose series at 6 and 14 weeks of age.^32^ The children who are age-eligible to receive rotavirus vaccinations are those born during or after mid-June 2009 and who were 6 weeks of age or older when the rotavirus vaccine was introduced in the EPI.

From April to July 2013, National Institute for Communicable Diseases (NICD), a division of the National Health Laboratory Service (NHLS) assisted with investigations of suspected diarrhoeal-illnesses outbreaks in two provinces: ZF Mgcawu District Municipality, Northern Cape province (NCP) and eThekweni Metropolitan Municipality, KwaZulu-Natal (KZN) province. This article describes the epidemiological, clinical and microbiological characteristics of the cases and the role of rotavirus infection in these outbreaks.

## Methods

### Case definition

Diarrhoeal-illness outbreak is defined as an increase in the number of diarrhoeal-illness cases more than what is normally expected in ZF Mgcawu District, NCP, 09 April–09 July 2013, and in eThekwini Metropole, KZN province, 01 May and 31 July 2013. A case was defined as the passage of ≥ 3 loose or watery stools in a 24-h period, with or without vomiting, fever and abdominal cramps between 09 April and 09 July 2013 in ZF Mgcawu District, NCP, and 01 May and 31 July 2013 in eThekwini Metropole, KZN province.

### Study design and study population

A descriptive cross-sectional study was conducted. The study population included all patients presenting for diarrhoea treatment at public and private healthcare facilities and fulfilling the case definition.

### Epidemiological investigation

The diarrhoeal-illness outbreak in NCP was confirmed by comparing the numbers of diarrhoea cases reported to NCP Department of Health by ZF Mgcawu District in 2012 and 2013. A similar approach was used in the KZN province. In both outbreaks, active case finding at healthcare facilities was initiated with a recommendation for stool sample collection from case-patients. Diarrhoea cases were identified by reviewing healthcare facilities (hospitals and clinics) records and line-listing patients with a diagnosis of diarrhoea or acute gastroenteritis. After obtaining consent, case investigation forms (CIFs) were completed by interviewing parents or guardians or caregivers during the households’ visits. Data collected included patient demographic information (name, age, sex, physical address), clinical features (symptoms and diagnosis), disease outcomes (discharged, hospitalised, death) and risk factors such as rotavirus vaccination history, occupational history and crèche or school (primary, secondary or tertiary level) attendance. Rotavirus vaccination history was sourced from the Road to Health Card (RTHC). Selected healthcare facilities were visited to investigate case management and infection control practises.

### Laboratory investigation

#### Stool specimens

Eighty-eight stool specimens from NCP and 242 from KZN province were tested at Centre for Enteric Diseases (CED), NICD/NHLS using enzyme immunoassays (EIAs), genotyping and sequencing for the detection and characterisation of rotavirus strains according to standardised methods. Nucleotide sequence analysis and phylogenetic comparisons of selected VP7 genes (1062 bp) were performed using MEGA X.^33^ The evolutionary history was inferred using the Neighbor-Joining method.^34^ The evolutionary distances were computed using the Tamura 3-parameter method.^35,36^ The percentage of replicate trees in which the associated taxa clustered together in the bootstrap test (1000 replicates) is shown next to the branches.^37^ Real-time reverse transcription polymerase chain reaction (RT-PCR) and real-time PCR detection of other enteric viruses were also performed according to standard protocols.

A subset of stool specimens (*n* = 61) from NCP was tested for enteric bacterial and parasitic infections at CED and the Centre for Healthcare-Associated Infections, Antimicrobial Resistance and Mycoses (CHARM), respectively. Enteric bacterial pathogens were tested using routine microscopy, culture and sensitivity screening (MCS) at the NHLS laboratory in Upington and CED. Additional RT-PCR methods were also performed at CED to type enteric bacteria detected. Calcofluor and trichrome staining for Microsporidia; Modified Ziehl-Neelsen staining for *Cryptosporidium* spp., *Cyclospora* spp. and *Isospora* spp.; concentration and direct wet preparations for detection of protozoan cysts or trophozoites or oocyts and helminth eggs or larvae were performed at CHARM. No bacterial or parasitic testing was performed on the KZN stool specimens.

#### Water samples

Water samples were collected at ZF Mgcawu District Municipality water reticulation system at two residential areas with the most reported diarrhoeal cases. Samples were sent to the Enteric Viruses and Environmental Virology Research group, Department of Medical Virology, University of Pretoria. Microbiological analysis was performed according to South African National Standards (SANS) 241-1, (2011) for drinking water. Samples were tested for faecal contamination indicators (faecal coliforms and total coliforms) and *E. coli*. In addition, samples were tested for the presence of epidemic-prone enteric viruses using RT-PCR as described previously.^38^ No water samples were collected at eThekwini outbreak.

### Data management and statistical analysis

Microsoft Office Excel 2007 and Epi-info version 7 were used for data management and analysis. Data cleaning was performed to check for duplicates, miscoded and out of range values (e.g. incorrect data entry such as date of birth, date of disease onset and date of hospital or clinic visits or admission). Descriptive statistical analysis was carried out and results expressed as absolute numbers and percentages.

### Ethical consideration

Ethical clearance was obtained from the University of the Witwatersrand, Human Research Ethics Committee (Medical) (M110499). Informed consent was obtained from parents or guardians to conduct interviews.

## Results

### ZF Mgcawu District Municipality, Northern Cape province

#### Epidemiological findings

**FIGURE 1:**
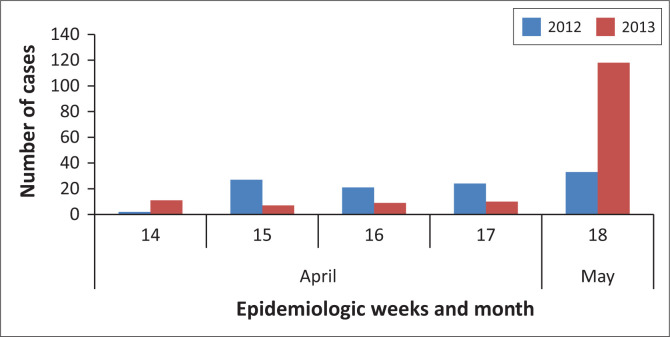
Distribution of diarrhoeal-illness cases during epidemiologic weeks 14–18 in 2012 and 2013, ZF Mgcawu District Municipality, Northern Cape province.

**FIGURE 2:**
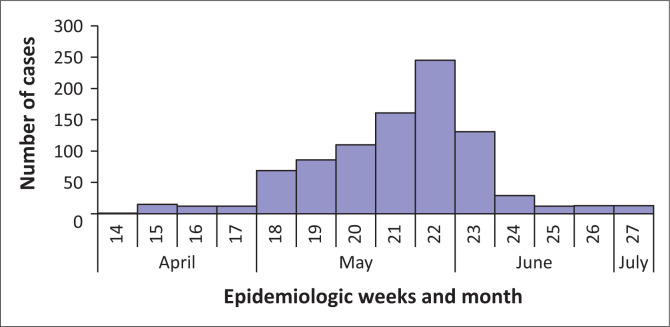
Distribution of diarrheal-illness cases by epidemiological week and month of disease onset, 09 April–09 July 2013, ZF Mgcawu District Municipality, Northern Cape province.

**FIGURE 3:**
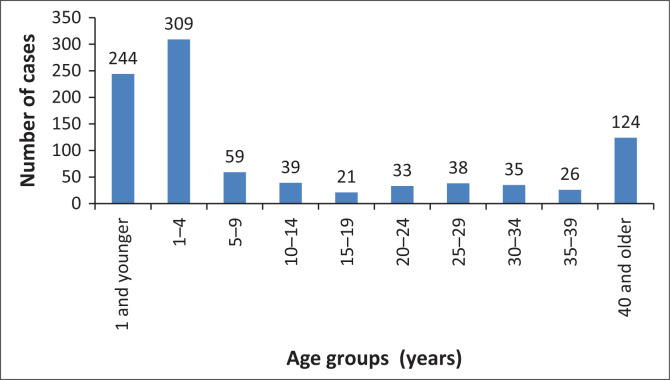
Diarrhoeal cases distribution per age groups in years, 09 April–09 July 2013, ZF Mgcawu District Municipality, Northern Cape province.

Approximately 953 diarrhoeal-illness cases were recorded from 09 April to 09 July 2013 (epidemiologic weeks 18–28) from 32 healthcare facilities (including public and private healthcare facilities). A comparison of the number of diarrhoeal-illness cases reported in week 18 of 2013 in ZF Mgcawu District Municipality with the same period in 2012 revealed a more than threefold increase in the number of cases (Figure 1). The peak number of cases was recorded during epidemiologic week 22 (week starting 27 May 2013), but declined to low levels by mid-June 2013 (Figure 2). Children < 5 years of age accounted for a high proportion of cases (58%, 553/953), and the majority (44%, 244/553) of whom were < 1 year of age (Figure 3). Of the rotavirus positive cases with age recorded (*n* = 37), 86% (32/37) were children < 5 years, while 57% (21/37) were < 1 year old.

Where diseases outcome was known (*n* = 847), 9% (76/847) of case-patients required hospitalisation, of whom 60% (46/76) were paediatric admission and 93% (43/46) were children < 5 years. In total 88% (747/847) of case-patients visited the outpatient department for treatment. In addition, 0.2% (2/847) cases-patients refused hospital treatment or absconded, 3% (22/847) of case-patients consulted at local clinics and were referred to district hospital for further treatment; however, hospital outcome remained unknown. Six diarrhoea-associated deaths were reported, with a 0.6% (6/953) outbreak case fatality rate (CFR); all deaths were in children ≤ 18 months old treated at the district hospital. Rotavirus testing was performed in one of the diarrhoea-associated deaths and the results were negative.

#### Laboratory findings

**Stool specimens:** In total, 88 stool specimens were tested at CED, NICD-NHLS. Rotavirus was detected in 43% (38/88) of specimens, while other enteric viruses (adenovirus, norovirus GI and GII, astrovirus, sapovirus and bocavirus) were detected in a further 19 cases (22%), mostly occurring as co-infections with rotavirus (63%, 12/19).

**FIGURE 4:**
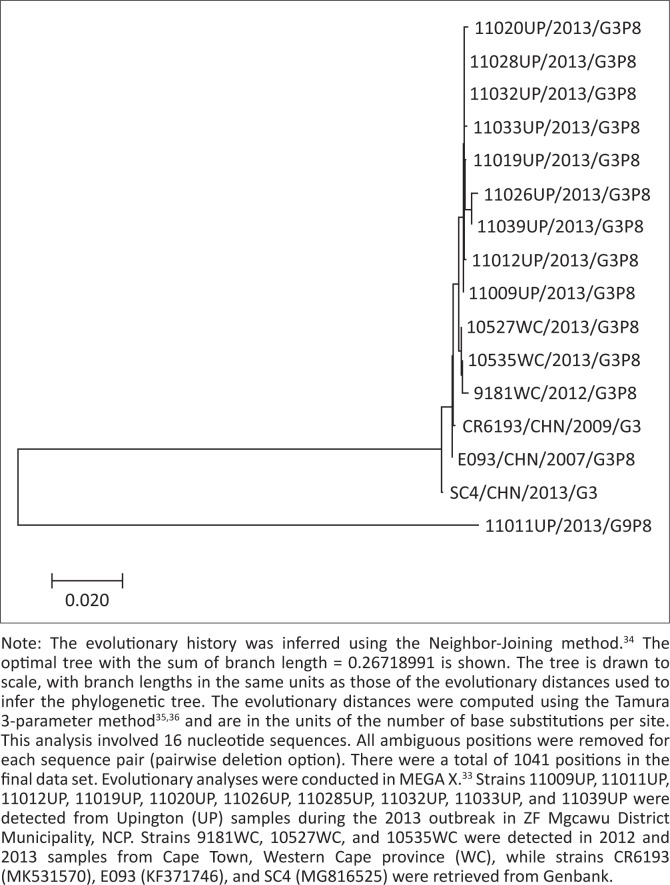
Phylogenetic tree of the VP7 gene, genotype G3 strains, 09 April–09 July 2013, ZF Mgcawu District Municipality, Northern Cape province.

Genotype G3P[8] (45%, 17/38); G9P[8] (42%, 16/38); G2P[6] (11%, 4/38) and G1P[8] (3%, 1/38) strains were circulating during the outbreak. Phylogenetic analyses of the VP7 gene of selected rotaviruses showed that the G3 strains were not markedly different from those circulating in South Africa during 2012 and 2013 (Figure 4).

Enteric bacterial pathogens such as diarrhoeagenic *E. coli*, non-typhoidal *Salmonella* (NTS), *Shigella* spp. and *Aeromonas* spp. were isolated in 26% (16/61) of the stool specimens submitted for screening, with rotavirus co-infection in 38% (6/16).

**Water samples:** No enteric pathogens were detected in the water samples.

#### Vaccination status of rotavirus positive cases

Interviews were conducted with 53% (20/38) of the rotavirus positive cases. Of the cases interviewed, 85% (17/20) were age-eligible to receive rotavirus vaccination, with a further 88% (15/17) age-eligible to receive two vaccine doses and two children (12%, 2/17) age-eligible to receive one rotavirus vaccine dose. A total of 47% (7/15) of the rotavirus positive cases age-eligible to receive two doses of rotavirus vaccine had been vaccinated (Table 1). The two cases age-eligible to receive one vaccine dose had received it; however, both cases developed diarrhoea ≤ 14 days after vaccination (range: 0–6 days after vaccination). The vaccination status of the diarrhoea-associated deaths could not be ascertained.

**TABLE 1:** Vaccination status of rotavirus cases, ZF Mgcawu District and eThekwini Metropolitan.

Vaccination history†	ZF Mgcawu District (NCP)		eThekwini Metropolitan (KZN)	
	Number of cases	%	Number of cases	%
Total rotavirus positive cases	38	-	134	-
Total cases interviewed	20	53	28	21
Number of cases age-eligible for rotavirus vaccine (date of birth ≥ mid-June 2009 and ≥6 weeks of age)	17	85	20	71
Age-eligible for two doses	15	88	18	90
Age-eligible for one dose	2	12	2	10
Number of cases age-eligible for two doses	15	-	18	**-**
Received two doses	7	47	17	94
Received one doses	4	27	1	6
No doses received	1	7	0	-
Unknown doses received	3	20	0	-
Number of cases age-eligible for one dose	2	-	2	**-**
Received one dose	2^b^	100	2‡	100
No doses received	0	-	0	-

NCP, Northern Cape province; KZN, KwaZulu-Natal.

† , Proof of vaccination obtained from Road to Health Card;

‡ , Both cases developed disease within ≤14 days after vaccination.

All the formal settlements, where most of the cases were reported, had indoor and/or outdoor municipal tap water, flushing toilets and municipal refuse removal. In diarrhoea cases reported from informal settlements, patients had communal municipal tap water, municipal refuse removal and bucket system toilets.

### eThekwini Metropolitan Municipality, KwaZulu-Natal province

#### Epidemiological findings

**FIGURE 5:**
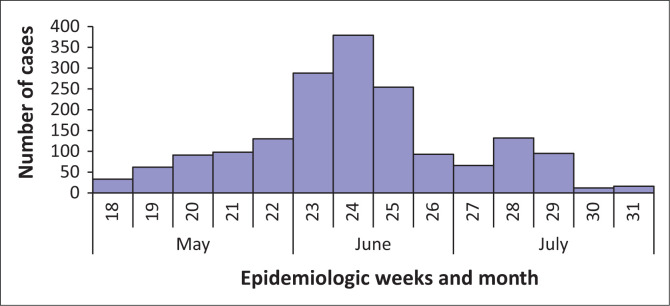
Distribution of diarrheal-illness cases by epidemiological week and month of disease onset, 01 May–31 July 2013, eThekwini Metropolitan Municipality, KwaZulu-Natal.

In total, 1749 cases of diarrhoea were recorded from 01 May to 31 July 2013 (epidemiologic weeks 18–31) from 61 healthcare facilities (including public and private healthcare facilities). Peak case numbers were recorded during epidemiologic week 24 (week of 09–15 June 2013), but declined towards end of July 2013 (week starting 21 July 2013) (Figure 5).

When age was recorded, children < 5 years of age accounted for a higher proportion of cases (95%, 1586/1661), particularly those < 1 year (47%, 788/1661). Of the rotavirus cases with age recorded, 65% (68/104) were < 1 year old. Twenty-six diarrhoea-associated deaths with 1.5% (26/1749) CFR were reported from three public hospitals, and the majority (81%, 21/26) were children aged < 1 year. The deaths occurred within 12 h of presentation to hospital. Rotavirus testing was performed in two cases who demised, one of whom was rotavirus positive. The vaccination status for the diarrhoea-associated deaths could not be ascertained.

#### Laboratory findings

**Stool specimens:** Stool from 242 case-patients was tested at CED, NICD/NHLS. Rotavirus was detected in 55% (134/242) of specimens. Other enteric viruses were also detected (30%, 73/242 occurring mostly as mixed infections with rotavirus (51%, 37/73).

**FIGURE 6:**
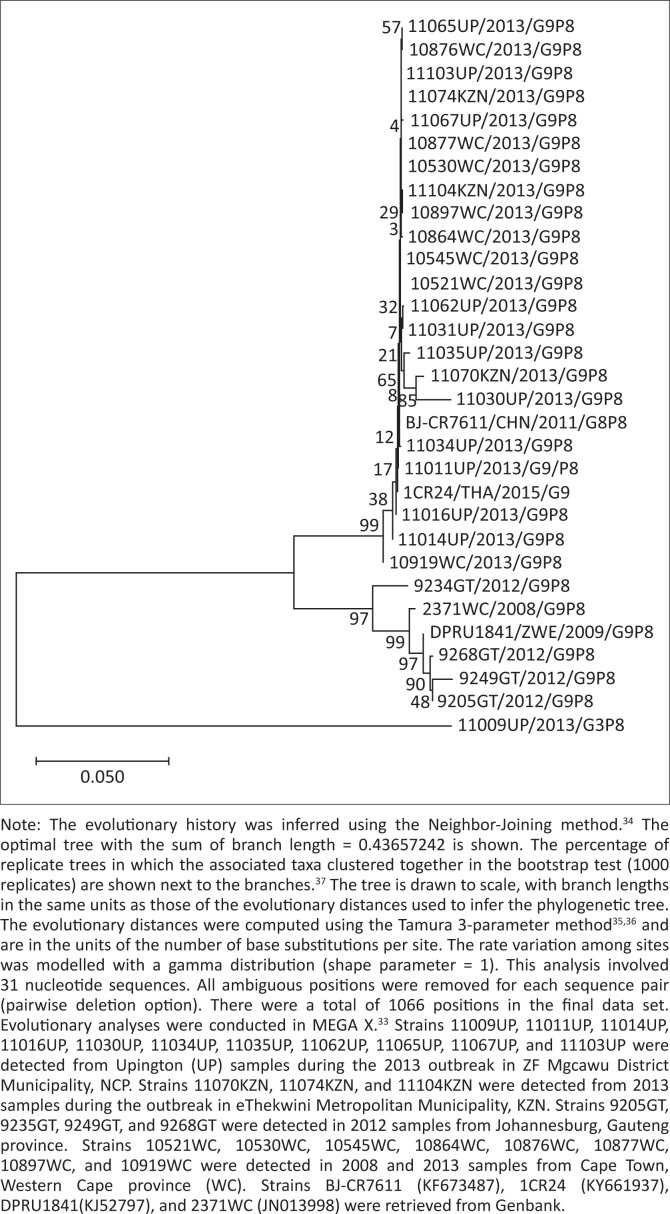
Phylogenetic tree of the VP7 gene, genotype G9 strains, 01 May–31 July 2013, eThekwini Metropolitan Municipality, KwaZulu-Natal.

Genotype G2P[4] and G9P[8] were the most common rotavirus strains identified (54%, 72/134 and 39%, 52/134, respectively). Phylogenetic analyses of the VP7 gene and G9 strain indicated that the 2013 G9 strains detected in NCP and KZN outbreaks were not markedly different from those circulating in South Africa during 2012 and 2013 (Figure 6).

#### Vaccination status of rotavirus positive cases

Of the rotavirus positive cases interviewed (*n* = 28), proof of vaccination status was available for 71% (20/28) who would have been age-eligible to receive one or two doses of rotavirus vaccine. The majority (94%, 17/18) of cases age-eligible to receive two rotavirus vaccine doses had received the vaccine; one case had only received one dose. Of the two cases age-eligible to have received one dose, both had received it, and both developed diarrhoea ≤ 14 days after vaccination (Table 1).

## Discussion

The increase in diarrhoea cases reported in both ZF Mgcawu District (NCP) and eThekwini Metropolitan Municipality (KZN) coincided with the annual rotavirus season in South Africa in 2013. Rotavirus was detected as co-infections with other enteric viruses and bacteria in some cases. This made the interpretation of the outbreak data difficult as we expect an increase in diarrhoea cases in children < 5 years during the cool, dry winter months. However, in the 2013 rotavirus season, diarrhoea cases were higher than previous seasons in 2009–2012.^39^ This increase was observed despite universal rotavirus vaccination being available and warranted additional investigation.

There are several possible reasons for the increase in diarrhoea-associated hospitalisations and the predominance of rotavirus detection in the two outbreaks. These include suboptimal rotavirus vaccine coverage, delayed vaccination and reduced rotavirus vaccine efficacy. During the 2013 outbreaks, children with rotavirus whose vaccination history was obtained and who were eligible to receive two doses of rotavirus vaccine, 73% (24/33) had received the vaccine on time (Table 1). These figures agree with the WHO-UNICEF rotavirus vaccine coverage estimates (71%) for 2013, but are below the official rotavirus vaccine coverage estimates (89%) reported by the South African National Department of Health.^40^ Furthermore, there may be regional differences in vaccine coverage as only 47% (7/15) of the children in NCP received their rotavirus vaccine, while 94% (17/18) in KZN received their rotavirus vaccines according to the prescribed schedule (Table 1).

The high rotavirus vaccine coverage seen in children interviewed in the KZN outbreak was concerning. However, a correlation has been shown between the timing of rotavirus season onset and the birth rate for different geographic regions in the United States.^41^ It has been suggested that in regions with higher birth rates, faster growth of the population of susceptible infants through new births may result in smaller observed declines in the rate of diarrhoea-associated hospitalisations than in regions with lower birth rates.^42^ These results may partially explain the observations in KZN. Further investigation on the impact of district-specific trends (birth rate and age of birth cohorts, etc.), geographic and socio-economic variations at national and sub-national levels on rotavirus transmission dynamics and subsequent effects of vaccination may be necessary.

One of the limitations of the investigation was the limited information on rotavirus vaccination history obtained during the outbreak (53% in NCP and 22% in KZN). This was because of the inability to locate and interview cases with caregivers often providing inaccurate contact details and residential addresses, or no one available at the house during the household visit. Future diarrhoeal-illness outbreak investigations, especially in children < 5 years, need to improve data collection on rotavirus vaccination to provide a clearer picture on the impact of the vaccine on rotavirus diarrhoea and associated hospitalisation.

Rotavirus vaccines provide homotypic and broad heterotypic protection and effectively prevent severe rotavirus diarrhoea, but are less efficacious against mild rotavirus diarrhoea or asymptomatic rotavirus infection.^10^ A randomised, placebo-controlled multicentre trial conducted in South Africa and Malawi showed vaccine efficacy against severe rotavirus gastroenteritis of 76.9% and efficacy against all-cause severe gastroenteritis of 44.1% for the South African infant cohort.^27^

Because the rotavirus vaccine was introduced into the South African public immunisation programme in 2009, rotavirus-specific hospitalisation decreased by 54% – 58% in 2010 and 2011 and there was a one-third reduction in total diarrhoea hospitalisations in children < 5 years.^43^ Another study in Soweto, South Africa, showed that all-cause diarrhoea hospitalisations in children < 5 years temporally decreased by 34% – 57% in the post-rotavirus vaccination era.^44^ In addition, rotavirus vaccine effectiveness against rotavirus diarrhoea hospitalisation was estimated at 57% (95% confidence interval [CI], 40–68) for two doses.^19^ A trend towards delayed rotavirus season onset because of the introduction of rotavirus vaccine in August 2009 has been observed.^43,45^

Vaccine efficacy varies across socio-economic settings, being lower in low- and middle-income countries.^46^ The reasons are probably multifactorial; putative factors include nutrition (malnutrition, micronutrient deficiencies), the presence of competing enteric viruses or helminths, co-administration of other oral vaccines (i.e. oral polio vaccine [OPV]), the presence of maternal antibodies and the balance between age of natural rotavirus infection and the timing of vaccination.^10,46,47^

Heterotypic immunity afforded by the Rotarix^®^ vaccine should provide protection against the strains detected in both outbreaks. The rotavirus strains detected in both outbreaks are common strains circulating globally, in keeping with natural rotavirus strain variation, and no evidence of the emergence of a novel rotavirus strain.^48^ Similarly, post-vaccination surveillance data from United States, Australia and Latin America did not indicate that rotavirus vaccines are exerting strain selection pressure.^49^

Of the diarrhoea-associated deaths reported in the diarrhoea outbreaks (six in NCP and 26 in KZN), three cases (9%, 3/32) were screened for rotavirus and one case was rotavirus positive. Because of insufficient data, it cannot be established how many of the deaths could be attributed to rotavirus. Future outbreak investigations should concentrate on obtaining specimens from patients who died to establish rotavirus-associated CFRs and help to elucidate causation. However, late hospital presentation, a lack of oral rehydration solution (ORS) at clinics and complacency by healthcare workers because of the success of the rotavirus vaccine in previous seasons could have led to the deaths associated with the 2013 diarrhoea season.

Other limitations included raised awareness among healthcare workers and communities, improved active diarrhoea case finding leading to increased case reporting, an increase in the number of specimens submitted for rotavirus testing and failure to screen all stool specimens from KZN outbreak for bacteria and parasites, few stool specimens collected and tested for rotavirus from NCP outbreak.

## Conclusion and recommendations

Investigation showed a prominence of rotavirus with no evidence of the emergence of novel rotavirus strains. While these events represent true diarrhoea outbreaks in that the number of cases increased in comparison to 2011 and 2012 diarrhoea seasons, fewer cases were reported in comparison to the pre-rotavirus vaccine years.

In conclusion, the existence of diarrhoea outbreaks in NCP and KZN provinces was confirmed. The predominance of rotavirus during the 2013 outbreaks is of concern. However, the diarrhoea cases were community-acquired and coincided with the annual rotavirus season in South Africa. Vaccination is aimed at decreasing severe diarrhoeal illness as opposed to preventing infection and mild disease. As a result, rotavirus cases will be seen particularly during rotavirus season. There is insufficient information on immunisation coverage and the associated rotavirus transmission dynamics to recommend changes to the current control measures against rotavirus disease in South Africa.

The investigation of these outbreaks highlighted several important points. There is a need to improve diarrhoea surveillance at all levels of healthcare facilities, including the private healthcare facilities. Data collection and management processes should be improved at healthcare facilities and health district levels to ensure good quality data for future analysis. Gaps in rotavirus vaccine coverage should be addressed through stock management as well as healthcare worker education and awareness. Health promotion activities within communities are recommended, especially prior to the rotavirus season, and should target education on diarrhoea prevention, the use of ORS in children with diarrhoea, recognising the signs of dehydration (when to seek medical care) and childhood immunisation promotion. Appropriate diarrhoea management guidelines or algorithms must be available (oral rehydration corners) and easily accessible at all facilities. Management of acute diarrhoea at healthcare facilities should be improved and include stool specimen collection for laboratory analysis during outbreaks. Lastly, improved communication and collaboration between all stakeholders are vital to ensure maximum efficiency for control measures implemented.
