# Erratum: Investigation of two suspected diarrhoeal-illness outbreaks in Northern Cape and KwaZulu-Natal provinces, South Africa, April–July 2013: The role of rotavirus

**DOI:** 10.4102/sajid.v39i1.597

**Published:** 2024-03-15

**Authors:** Andronica M. Shonhiwa, Genevie Ntshoe, Noreen Crisp, Ayo J. Olowolagba, Vusi Mbuthu, Maureen B. Taylor, Juno Thomas, Nicola Page

**Affiliations:** 1 Division of Public Health, Surveillance and Response, National Institute for Communicable Diseases, National Health Laboratory Service, Sandringham, Johannesburg, South Africa; 2 School of Health Systems and Public Health, Faculty of Health Science, University of Pretoria, Pretoria, South Africa; 3 Communicable Disease Control, Department of Health, Kimberley, South Africa; 4 Communicable Disease Control, eThekwini Metropolitan Municipality Department of Health, Durban, South Africa; 5 Department of Medical Virology, Faculty of Health Sciences, University of Pretoria, Pretoria, South Africa; 6 National Health Laboratory Service, Tshwane Academic Division, Pretoria, South Africa; 7 Centre for Enteric Diseases, National Institute for Communicable Diseases, National Health Laboratory Service, Sandringham, Johannesburg, South Africa

In the published article, Shonhiwa AM, Ntshoe G, Crisp N, et al. Investigation of two suspected diarrhoeal-illness outbreaks in Northern Cape and KwaZulu-Natal provinces, South Africa, April–July 2013: The role of rotavirus. S Afr J Infect Dis. 2020;35(1),a159. https://doi.org/10.4102/sajid.v35i1.159, there was an error in the spelling of the last authors name, instead of Nicole A. Page it should read Nicola Page. There was also and error in the labelling in Figure 3.

The age group for under 1 year cases is wrongly labelled in the final published article.

Instead of:

1 and younger

**FIGURE 3:**
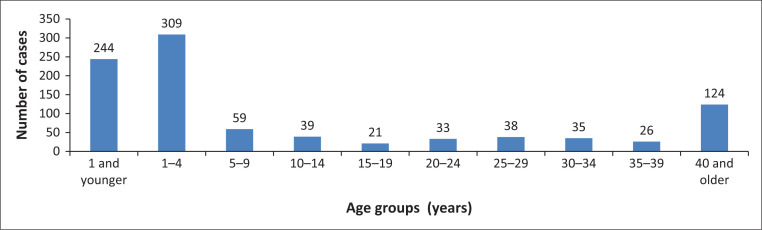
Diarrhoeal cases distribution per age groups in years, 09 April–09 July 2013, ZF Mgcawu District Municipality, Northern Cape province.

It should be:

< 1.

**FIGURE 3:**
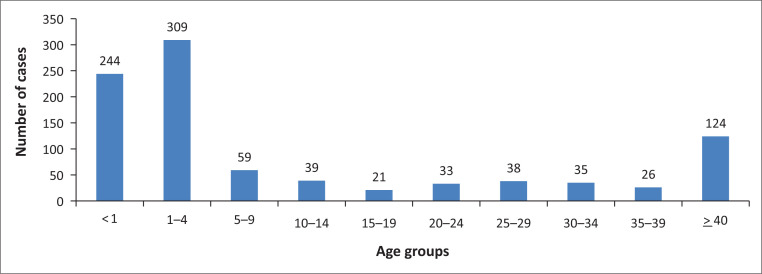
Diarrhoeal cases distribution per age groups in years, 09 April–09 July 2013, ZF Mgcawu District Municipality, Northern Cape province.

The publisher apologises for this errors. The correction does not change the study’s findings of significance or overall interpretation of the study’s results or the scientific conclusions of the article in any way.

